# Human papillomavirus infection & anal cytological abnormalities in HIV-positive men in eastern India

**DOI:** 10.1186/s12879-018-3618-3

**Published:** 2018-12-27

**Authors:** Abhilasha Gautam, Jaya Chakravarty, Vijay Kumar Singh, Amrita Ghosh, Shashi Bhushan Chauhan, Madhukar Rai, Shyam Sundar

**Affiliations:** 1 Department of Medicine, Institute of Medical Sciences, Banaras Hindu University, Varanasi, 221005 India; 2 Department of Pathology, Institute of Medical Sciences, Banaras Hindu University, Varanasi, 221005 India

**Keywords:** HPV, HIV, Anal cytology, PCR, Anal cancer, India

## Abstract

**Background:**

Oncogenic Human papillomavirus (HPV) infections are closely associated with anal cancer which is high among human immunodeficiency virus (HIV) infected males. There are no data regarding anal HPV infection and cytological abnormalities in HIV positive males receiving free therapy in the national program. Thus, this cross-sectional study was performed to assess the prevalence and risk factors of anal HPV infection and cytological abnormalities in HIV positive males.

**Methods:**

We screened 126 HIV-positive male patients attending the antiretroviral treatment center (ART) between 2014 and 2015 with anal papanicolaou smear cytology and HPV-DNA testing. HPV-DNA was detected by using polymerase chain reaction (PCR) method with two consensus primer sets E6 and MY09/11 and further analyzed for the presence of various HPV genotype by Sanger sequencing. Risk factors associated with anal cytological abnormalities and HPV infection was analyzed by using univariate and multivariate logistic regression models.

**Results:**

Out of 126, 52 were on antiretroviral therapy. 91% were married to female partners but during the study 48 (38%) gave positive history of anal intercourse with other men. Anal cytology was done in 95 patients, out of which 60 (63.15%) had cytological abnormalities. LSIL (low-grade squamous intraepithelial lesions) was present in 27 (45%), ASCUS (atypical squamous cells of undetermined significance) in 31 (52%) and ASC-H (atypical squamous cells cannot exclude a high-grade squamous intraepithelial lesion) in 2 (3.33%). In multivariate analysis, the risk factors for cytological abnormality were presence of history of anal intercourse (OR, 6.1; 95% CI, 2.0–18.7) and WHO stage III & IV (OR, 2.7; 95% CI, 1.1–7.5). HPV-DNA was detected in 33/119 (27.73%) patients. The most prevalent HPV type in the study was HPV-16 (10.08%), other HPV types detected were 18,31,35,17,66,72,52,68 and 107 (17.65%).

**Conclusions:**

High prevalence of anal cytological abnormalities in our study suggests that regular anal Pap smear screening should be done in HIV positive males in the ART center.

## Background

With the scale-up of antiretroviral therapy and longer life expectancy of HIV infected individuals, there is a growing concern about infection-related cancers. Anal cancer is one of the frequent non-AIDS-defining malignancies in the HIV-infected population [1]. In a large study involving 13 cohorts from North America, the unadjusted anal cancer incidence rates per 100,000 person-years were 131 for HIV infected MSM (men having sex with men), 46 for other HIV-infected men, and 2 for HIV-uninfected men [2]. High-grade squamous intraepithelial lesions (HSIL) are considered premalignant and may progress to anal cancer. Risk factors for the development of anal squamous intraepithelial lesion system (SIL) are anal HPV infection, high-risk sexual behavior and HIV infection, especially with lower CD4 levels [3, 4]. HPV infection is known to be a causative factor for both anal SIL and anal cancer [5–7].

The age-standardized rates (ASR) of anal cancer in different regions of India varies from 0.1–0.7 per 100,000 men per year [8]. A study from an HIV cancer clinic in India showed that the proportional incidence ratio in HIV-positive males for anal cancer was 10.3 [9]. Despite the known high risk of anal cancer in HIV positive males, there are very few studies in India regarding the prevalence of HPV and anal cytological abnormalities in this population. A small study among HIV positive and negative MSM in India showed that 27.7% had abnormal anal cytology which was increased in HIV-positive men as compared to HIV-negative men (35% versus 20%, *p* = 0.180) [10]. In another large study of HIV positive MSM in India, the prevalence of oncogenic anal HPV was 49% [11]. Most of the studies of anal cytological abnormalities and HPV infection have focused on MSM, however, other HIV positive men are also at an increased risk of anal cancer. As of September 2014, 1.88 million PLHIV (people living with HIV/AIDS) have been registered in 453 National AIDS control organization (NACO) run ART centers in India, 60% of whom are male patients [12]. Despite the large number of males in the program, there are no studies on anal cytological abnormalities and HPV infection in these patients. Thus, this study was done to assess the prevalence and risk factors of anal HPV infection and anal cytological abnormalities in HIV positive males attending the ART center.

## Methods

### Study site

This cross-sectional exploratory study was conducted between 2014 and 2015 in HIV positive men attending the ART Center. The ART center of Banaras Hindu University (BHU) is one of the largest center in the Eastern part of the country and has approximately 15,000 PLHIV registered in it.

### Study population and sample collection

The study was approved by the Ethics committee of Institute of Medical Sciences, Banaras Hindu University (EC registration No- ECR/526/Inst./UP/2014 Dt-31.1.14). All HIV positive men above the age of 18, attending the ART centers were eligible for the study. A written informed consent was obtained from all the participants. A detailed history including sexual history was obtained. Baseline demographic data was collected from the cards of the patient maintained in the ART center. CD4+ lymphocyte counts were determined using BD FACS Calibur (FACS Calibur Becton Dickinson Biosciences) and the values were expressed as cells/μL.

Anal samples were taken from the anal squamous-columnar junction of the anal canal. These samples were than separated into two parts, initially a smear was prepared for anal cytology screening and immersed immediately in 95% alcohol and the other anal scrape sample was collected in 15 ml falcon tube containing 2 ml 1X phosphate buffered saline (PBS) which was stored at − 20 °C for DNA extraction and PCR amplification. Cytology smears were evaluated by a pathologist under light microscope and cytological changes were classified according to Bethesda system normal i.e. no cell changes; NILM (negative for intraepithelial lesions and malignancy) which includes inflammatory changes, organisms, atrophic changes and reactive changes; ASCUS (atypical squamous cells of undetermined significance) ; ASC­H (atypical squamous cells cannot exclude a high-grade squamous intraepithelial lesion) ; LSIL (low-grade squamous intraepithelial lesions) and HSIL (high-grade squamous intraepithelial lesions) [13].

### DNA extraction and HPV-DNA detection

HPV-DNA were extracted from epithelial cell suspension by using QIAamp DNA Blood Mini Kit (QIAGEN, Hilden, Germany) according to manufacturer’s protocol, isolated DNA were dissolved in 80ul milli-Q water. Quality and quantity of DNA were checked in NanoDrop Spectrophotometers (Thermo Scientific, USA). After validating the DNA extraction results, samples were stored at -20^o^ C until further processing. To detect HPV, Polymerase chain reaction (PCR) reaction (amplification) was performed by using two different consensus primer sets, E6 consensus primer targeting a 250-bp oncogenic region of the HPV E6 ORF (Open reading frame) [14] and MY09/11 consensus primer targeting a 450-bp region of the HPV L1 ORF in HPV genome [15].

PCR products were viewed by Agarose Gel Electrophoresis (AGE) during optimization procedures, and as needed. 1.4% agarose gels were prepared for electrophoresis.

### Genotyping by sequencing

All HPV positive PCR products were subjected to sequencing for confirmation of HPV types. DNA sequencing was performed on 3730XL DNA genetic analyzer (Applied Biosystem, USA) using the Big Dye Terminator Cycle Sequencing Ready Reaction Kit version 3.1 (Applied Biosystem, CA, USA) with the E6 and MY09/11 consensus primers by SciGenom, Labs Pvt. Ltd., Cochin, Kerala, India. The resulting sequences were compared with HPV sequences of known types using the basic local alignment search tool (BLAST) from the National Centers for Biotechnology Information (NCBI) website (https://blast.ncbi.nlm.nih.gov). HPV types 16, 18, 31, 33, 35, 39, 45, 51, 52, 56, 58, 59, 68, 73 and 82 were considered as highly oncogenic high­risk (HR), while HPV types 26, 53 and 66 as probably oncogenic. HPV types 6, 11, 40, 42, 43, 44, 54, 61, 70, 72 and 81 were considered as low oncogenic potential low­risk (LR) [16].

### Statistical analysis

Statistical analysis was performed using the Statistical Package for Social Sciences (SPSS) software, version 16. For Bi-variable logistic regression analysis, variables were compared between HPV positive-negative and Pap smear cytology between abnormal-normal in HIV- positive men by performing chi-square test, t-test or the Mann-Whitney U-test where appropriate. Binary logistic regression analysis was performed to analyze the associations between variables. Results were estimated by means of odds ratio (OR) and 95% confidence intervals (CI). Two-tailed *P* values less than 0.05 were considered statistically significant.

## Result

### Baseline characteristics of cases

We screened 126 HIV-positive men in this study. Baseline characteristics of the HIV positive men are given in Table 1. The mean age was 35.37 ± 8.2 years, median CD4+ cell counts at baseline was 129/μL and at the time of sample collection was 253/μL. Mean weight and mean hemoglobin was 49.53 ± 8.45 Kg and 11.2 ± 1.73 g/dl respectively. 74 patients were treatment naive and 52 were on ART with a mean duration of treatment of 12 (9–22) months. The documented risk for HIV was heterosexual contact for 125 males in the records of the center and 91% were married to female partners. However, during the study 48 (38%) gave positive history of anal intercourse with other men.

**Table 1:** Baseline characteristics and risk factor analysis

Baseline characteristics		Risk factor analysis for abnormal and normal anal cytology in HIV positive men (*n* = 95)						Risk factor analysis for HPV positive and negative in HIV positive men (*n* = 119)			
		Frequency	Frequency	Uni-variate		Multi-variate		Frequency	Frequency	Uni-variate	
		No. (%)	No. (%)					No. (%)	No. (%)		
		Positive (*n* = 60)	Negative (*n* = 35)	OR	*P*-value	OR	*P*-value	Positive (*n* = 33)	Negative (*n* = 86)	OR	*P*-value
				(95% CI)		(95% CI)				(95% CI)	
Age (years)	< 35 year	27 (45.0)	18 (51.4)	0.7 (0.3–1.6)	0.54			15 (45.5)	41 (47.7)	0.9 (0.4–2.0)	0.82
		33 (55.0)	17 (48.6)	1				18 (54.5)	45 (52.3)	1	
Education	Illiterate	12 (20.0)	5 (14.3)	1.5 (0.4–4.6)	0.48			1 (3.0)	16 (18.6)	0.1 (0.0–1.0)	0.05
		48 (80.0)	30 (85.7)	1				32 (97.0)	70 (81.4)	1	
Employment	Unemployed	30 (50.0)	14 (40.0)	1.5 (0.6–3.4)	0.34			13 (39.4)	36 (41.9)	0.9 (0.3–2.0)	0.80
		30 (50.0)	21 (60.0)	1				20 (60.6)	50 (58.1)	1	
Urban/rural	Rural	45 (75.0)	26 (74.3)	0.9 (0.3–2.5)	0.93			26 (78.8)	61 (71.0)	0.6 (0.2–1.7)	0.38
		15 (25.0)	9 (25.7)	1				7 (21.2)	25 (29.0)	1	
Marital status	Married, Widowed, Divorced	6 (10.0)	1 (2.9)	0.2 (0.0–2.2)	0.22			6 (18.2)	4 (4.7)	0.2 (0.0–0.8)	0.22
	Never married	54 (90.0)	34 (97.1)	1				27 (81.8)	82 (95.3)	1	
History of active tuberculosis	Present	24 (40.0)	8 (22.9)	2.4 (1.1–6.1)	0.04			9 (27.3)	35 (40.7)	0.5 (0.2–1.3)	0.17
		36 (60.0)	27 (77.1)	1				24 (72.7)	51 (59.3)	1	
History of Alcohol intake	Yes	46 (76.7)	27 (77.1)	0.9 (0.3–2.6)	0.95			24 (72.7)	70 (81.4)	0.6 (0.2–1.5)	0.30
		14 (23.3)	8 (22.9)	1				9 (27.3)	16 (18.6)	1	
History of Smoking	Yes	48 (80.0)	28 (80.0)	1.0 (0.3–2.8)	0.99			26 (78.8)	72 (83.7)	0.7 (0.2–1.9)	0.52
		12 (20.0)	7 (20.0)	1				7 (21.2)	14 (16.3)	1	
History of Anal intercourse with other male.	Present	31 (51.7)	5 (14.3)	6.4 (2.1–18.7)	0.00*	6.1 (2.0–18.7)	0.00*	15 (45.5)	29 (33.7)	1.6 (0.7–3.7)	0.23
		29 (48.3)	30 (85.7)	1							
No. of Sexual partners	>one	50 (83.3)	27 (77.1)	1.5 (0.5–4.4)	0.39			26 (78.8)	70 (81.4)	0.8 (0.3–2.2)	0.74
		10 (16.7)	8 (22.9)	1				7 (21.2)	16 (18.6)	1	
History of hemorrhoid	Present	27 (45.0)	11 (31.4)	1.7 (0.7–4.2)	0.19			15 (45.5)	35 (40.7)	1.2 (0.5–2.7)	0.63
		33 (55.0)	24 (68.6)	1				18 (54.5)	51 (59.3)		
Anal symptoms (bleeding, discharge, pain, irritation)	Present	26 (43.3)	11 (31.4)	1.6 (0.6–4.0)	0.25			15 (45.5)	35 (40.7)	1.2 (0.5–2.8)	0.55
		34 (56.7)	24 (68.6)	1				18 (54.5)	51 (59.3)	1	
WHO HIV clinical staging	III + IV	30 (50.0)	9 (25.7)	2.8 (1.1–7.1)	0.02*	2.7 (1.1–7.5)	0.04*	14 (42.4)	37 (43.0)	0.9 (0.4–2.1)	0.95
	I + II	30 (50.0)	26 (74.3)	1				19 (57.6)	49 (57.0)	1	
CD4 count (ul) (at the time of study)	< 350	50 (83.3)	22 (62.9)	2.9 (1.1–7.7)	0.02*	1.6 (0.5–4.7)	0.37	26 (78.8)	61 (71.0)	1.5 (0.5–3.9)	0.38
		10 (16.7)	13 (37.1)	1				7 (21.2)	25 (29.0)	1	
Weight (kg)	< 40	7 (11.7)	6 (17.1)	0.6 (0.1–2.0)	0.45			4 (12.1)	10 (11.6)	1.0 (0.3–3.6)	0.94
		53 (88.3)	29 (82.9)	1				29 (87.9)	76 (88.4)	1	
Hemoglobin (g/dl)	< 10 mg/dl	14 (23.3)	7 (20.0)	1.5 (0.5–4.3)	0.70			7 (21.2)	19 (22.0)	0.9 (0.3–2.5)	0.91
		46 (76.7)	28 (80.0)	1				26 (78.8)	67 (78.0)	1	
Presence of anal wart on visual inspection	Present	14 (23.3)	7 (20.0)	1.5 (0.5–4.3)	0.70			7 (21.2)	21 (24.4)	0.8 (0.3–2.1)	0.71
		46 (76.7)	28 (80.0)	1				26 (78.8)	65 (75.6)	1	
ART status	Pre ART	32 (53.3)	19 (54.3)	0.9 (0.4–2.2)	0.92			23 (69.7)	48 (55.8)	1.8 (0.7–4.2)	0.17
	On ART	28 (46.7)	16 (45.7)	1				10 (30.3)	38 (44.2)	1	

### Prevalence and analysis of anal cytology

Out of 126 cases, 31 slides were broken, lost or found to be inadequate for result. Finally, 95 slides were examined out of which 60 (63.15%) had cytological abnormalities, of which 27 (45%) cases had LSIL, 31 (52%) had ASCUS and 2 (3.33%) had ASC-H. None of the patients had HSIL. 33 (34.73%) slides were negative for any cytological abnormalities and 2 (2.10%) had reactive inflammatory changes. In the univariate analysis risk factors for anal cytological abnormalities were history of anal intercourse (OR, 6.4; 95% CI, 2.1–18.7), with *p*-value of 0.00, history of active tuberculosis (OR, 2.4; 95% CI, 1.1–6.1), with p-value of 0.04, low CD4 count < 350/μl (OR, 2.9; 95% CI, 1.1–7.7) with p-value of 0.02, WHO clinical stage III + IV (OR, 2.8; 95% CI, 1.1–7.1) with p-value of 0.02. In multivariate logistic regression analysis, history of anal intercourse (OR, 6.1; 95% CI, 2.0–18.7) p-value 0.00 and WHO clinical stage (III + IV) (OR, 2.7; 95% CI, 1.1–7.5), p-value 0.04 were risk factor for anal cytological abnormalities given in (Table 1).

### HPV genotype distribution

Out of 126 cases enrolled in the study, 7 samples had inadequate DNA, therefore a total of 119 samples were examined out of which 33/119 (27.73%) were positive for HPV-DNA. Among men with history of anal intercourse the prevalence of HPV was 15/48 (31.25%) much higher than in heterosexual men where it was 18/78 (23%). In the univariate and multivariate logistic regression analysis no significant risk factor was identified for anal HPV detection by PCR (Table 1). All HPV- positive samples were subjected to direct sequencing method for confirmation of HPV types. It revealed 10 different high-risk (HR) HPV genotypes and all 33 HPV-positive sequences were recognized. HPV-16 the most prevalent HPV type in the study was, identified in (10.08%, 12/119) followed by HPV-31 (4.2%, 5/119), HPV-35 (3.36%, 4/119) HPV-18 (2.5%, 3/119), HPV-17, HPV-52, HPV-72 (1.6% each, 2/119) and HPV-66, HPV-68, HPV-107 (0.84% each, 1/119).

### Correlation between HPV-PCR and anal cytology

The overall prevalence of HR­HPV was 27.73% among the HIV­ positive men.

Among those with NILM, ASCUS, and LSIL, 24.24, 28.57 and 44.44% respectively were HPV positive (Table 2).

**Table 2:** Correlation between results of HPV-PCR and Anal cytology

HPV-PCR^a^ (*n* = 119)	Anal Cytology^b^				
	Normal cytology (n = 33)	ASCUS (*n* = 28)	ASC-H (n = 2)	LSIL (*n* = 27)	Inadequate Smear (*n* = 29)
HPV Positive 33 (27.73%)	8 (24.24%)	8 (28.57%)	0	12 (44.44%)	5 (17.24%)
HPV Negative 86 (72.26%)	25 (75.75%)	20 (71.43%)	2 (100%)	15 (55.56%)	24 (82.76%)

NOTE: ^a^ HPV-PCR was done in 119 samples as 7 patients with inadequate DNA. Among these 7 patients, 2 had normal cytology, 3 had ASCUS and 2 had inadequate smear

^b^Anal Cytology was performed in 95 samples

## Discussion

This is the first study among HIV positive men attending the ART center of the National program. An interesting finding in this exploratory cross-sectional study was that 38% of our study participants gave a history of anal intercourse with other men while in all except one participant the documented risk for HIV was heterosexual in the records of the center. In India due to fear of stigma, discrimination and criminalization of homosexuality many men fail to disclose their true sexual orientation. 91% of these men were either currently or previously married i.e. were or had been in heterosexual relationship. Studies have also shown that 23–42% of MSM are married to women in India thereby easily mistaken for being heterosexual [17–19].These findings in our study suggest that although bisexuality among HIV positive men in our set up is common, elicitation and documentation of this fact is poor.

However, the recent decriminalization of homosexuality in India will go a long way in improving disclosures regarding their sexual orientation.

Prevalence of HPV in HIV positive males in our study was 27.73%.This is similar to a study of HIV positive heterosexual males by Gandra et al., which showed the overall prevalence of HR-HPV and abnormal cytology to be 28% [20].While it was much lower than that observed in HIV-infected MSM in a meta-analysis of 53 studies, where the pooled prevalence of anal HPV infection was 93 and 74% for any type and any high-risk type, respectively [21]. Studies have shown that the HPV detection rate is critically affected by the choice of PCR primer sets [22]. This could be one of the reasons for the low prevalence of HPV in our population despite the fact that 38% of the participants were bisexual. Use of multiple primer sets would definitely increase the detection rate of HPV-DNA as observed in our unpublished data. As HPV types 16 and 18 causes nearly 90% of anal cancers, vaccination against HPV could be an effective strategy of preventing this malignancy. HPV vaccination is recommended for all immunocompromised patients up to 26 years of age and up to 40 years in MSM by European AIDS clinical society [EACS] and similar guidelines would also be relevant in India.

Abnormal anal cytology was present in 63.15% (60/95) in our study, 33% of them were also HPV positive. This is much higher than the 20–28% of cytological abnormality observed in recent studies in heterosexual HIV positive men [20, 23]. While it is similar (66–67%) to studies in HIV positive MSM [24]. Participants with poor WHO clinical stage and history of anal intercourse were also at risk for anal SIL similar to other studies [25]. Although none of the patients had HSIL, 27 had LSIL and 12 (44%) out of them were HPV positive. In a study that included 346 HIV-infected and 262 HIV-uninfected MSM, 62% of HIV-infected and 36% of HIV-uninfected men with LSIL at baseline progressed to HSIL [26]. This suggests that PLHIV with baseline cytological abnormality should be followed up to observe whether the lesions are progressing or not.

At present New York State screening (NYS) guidelines for anal cancer recommend that all HIV positive patients undergo annual visual and digital rectal exam plus an anal Pap screening for anal cancer. While the EACS recommends anal cancer screening for MSM and those with HPV associated dysplasia. In India, there are no recommendations for screening of HIV positive males as there is no data regarding the prevalence of anal cytological abnormality and HPV prevalence in HIV positive males. Anal cancer screening is a simple tool which could increase early detection of anal cancer in HIV positive males.

The limitations of our study were its cross-sectional nature, only one pathologist examined the slides, 31 slides count not be evaluated for cytology and the inability to detect multiple HPV types. Further, the results of our study cannot be generalized as these samples were only from HIV positive males in eastern India and studies from other part of the country needs to be done.

## Conclusions

This is the first study on anal cytological abnormalities and HPV infection among HIV positive males attending the National program in India. The prevalence of HPV infection in our study was 27.73%, which was much lower than HIV positive MSM but similar to that in HIV-negative MSM. At present there is no recommendation for HPV testing for HIV positive MSM or heterosexual males. However, HPV vaccination for PLHIV could be an effective strategy to decrease HPV related malignancy. 38% of subjects documented to be heterosexual in the ART center had a history of anal intercourse in our study thus efforts should be taken to elicit this history .

There was high prevalence of anal cytological abnormalities (63.15%) in our study subjects which was higher than studies of HIV positive heterosexual males and similar to HIV positive MSM. The main risk factor identified for abnormal anal cytology was history of anal intercourse and poor WHO clinical stage III&IV. Many guidelines recommend screening of PLHIV for anal cancer. In India there are no recommendations for screening of HIV positive males as there is no data regarding the prevalence of anal cytological abnormality in this cohort. Anal cancer screening as done for cervical cancer in women is a simple tool which could potentially reduce both incidence and mortality by allowing for early detection of anal cancer precursor lesions especially in HIV positive bisexual males.

## Abbreviations

ART: Antiretroviral therapy
ASC-H: Atypical squamous cells cannot exclude a high­grade squamous intraepithelial
ASCUS: Atypical squamous cells of undetermined significance
BHU: Banaras Hindu University
BLAST: Basic Local Alignment Search Tool
CI: Confidence intervals
COE: Center of Excellence
DNA: Deoxyribo nucleic acid
EACS: European AIDS clinical society
HIV: Human immunodeficiency
HPV: Human papillomavirus
HR: High-risk
LR: Low-risk
LSIL: Low-grade squamous intraepithelial lesions
MSM: Men having sex with men
NACO: National AIDS control organization
NCBI: National Centers for Biotechnology Information
NILM: Negative for intraepithelial lesions and malignancy
NYS: New York State screening
OR: Odds ratio
PBS: Phosphate buffered saline
PCR: Polymerase chain reaction
PLHIV: People living with HIV/AIDS
SIL: Squamous intraepithelial lesion system
SPSS: Statistical Package for Social Sciences
UPSACS: Uttar Pradesh State AIDS control society
